# Effects of using auto flow factor function on hemodialysis efficacy in regular hemodialysis patients

**DOI:** 10.1186/s12882-025-04508-2

**Published:** 2025-10-27

**Authors:** Said S. A. Khamis, Ahmed Ragheeb Tawfeek, Mohammed Fathy Ragab, Heba Kamal Abd elkhalk

**Affiliations:** 1 Department of Internal Medicine, Faculty of Medicine, University of Menoufia, Shebin El-Kom, Egypt; 2 Department of Internal Medicine, Ministry of Health, Menoufia Health Insurance Hospital, Shebin El-Kom, Egypt

**Keywords:** Auto flow factor, Dialysis, Dialysate flow, Heamodialysis

## Abstract

**Background:**

Recent HD systems have moved from pre-defined dialysate flow rates (e.g. 300, 500, 800 mL/min) to an individual setting, or an automatic adjustment with a fixed factor of the dialysate flow to the blood flow. Both allows more flexibility of choosing the appropriate dialysate flow as an optimal adjustment to the current blood flow.

**Aim of the work:**

To study the possible effects of using autoflow factor function on hemodialysis (HD) efficacy in regular hemodialysis patients.

**Methods:**

A prospective cross section study included 55 ESRD patients on hemodialysis and conducted in hemodialysis unit in Menoufia university hospital.

**Results:**

There was no statistically significant difference between the dialysate flow rate 500 mL/min and auto Flow factor regarding the Kt/V (*p* = 0.068).

**Conclusion:**

The present study demonstrates that the use of the Auto Flow Factor Function does not result in a statistically significant difference in dialysis efficacy compared to a fixed dialysate flow rate of 500 mL/min. Given the potential for dialysate and energy savings, the implementation of Auto Flow may represent a step toward environmentally sustainable dialysis without compromising patient care.

## Introduction

Hemodialysis (HD) is a technique which is used to achieve the extracorporeal removal of any waste products such as urea and creatinine and excess of water from the blood when the kidneys are in a condition of renal failure. HD is the most prevalent cause of modality of renal replacement therapy in patients with renal failure followed by kidney transplantation surgery and peritoneal dialysis [1].

The 1ry goal of hemodialysis is to maintain the intracellular and extracellular fluid environment that is characteristic of normal kidney function. This is followed by the transport of solutes such as urea & creatinine from the blood into the dialysate and by the removal of solutes such as bicarbonate from the dialysate to the blood. Solute concentration levels and molecular weight are the 1ry determinants of rates of diffusion. Small like molecules, such as urea diffuse so quickly, whereas larger molecules, as phosphate, β2- micro globulin, and albumin, and protein bound solutes diffuse more slowly. Diffusion, solutes pass through pores in the membrane by ultrafiltration “UF” [2].

During UF, there is no change in solute concentrations; its 1ry purpose is the removal of excess total body water. The patient’s physiological status should be assessed for each dialysis session, so that the dialysis prescription can be aligned with the goals for the session [3].

The novel AutoFlow (AF) function in dialysis machines, as 5008 Therapy System (Fresenius Medical Care, Bad Homburg, Germany), adjusts Qd automatically according to the effective rate of blood flow (Qb) of the patient at a selected AutoFlow factor (Qd = AF factor ¥ Qb) [4]. The consequence of this approach is a significant for saving in dialysis and decrease fluid consumption at the highest levels of achievable clearance which reached at an appropriately selected AutoFlow factor [5].

The automatic AutoFlow function adjusts the dialysate flow rate to the blood flow rate in order to enable dialysate savings compared to the standard dialysate flow rate setting [6]. Kidney Quality Initiative Clinical Practice Guidelines for Hemodialysis (HD) Adequacy, a low level of urea equilibrated Kt/V (eKt/V) of 1.2 is recommended for three dialysis per week and in the absence of residual renal function [7].

### Economic considerations

Although the primary target is to achieve the best treatment conditions and outcomes, improving patient and a constantly deteriorating health of patients beginning dialysis have resulted in economic restrictions for dialysis health care thereby necessitating a review of current practices. The dialysis dose delivered to a patient is influenced by a number of factors: first by the blood flow rate, the selected level of dialyzer, the treatment time, the treatment methods and the dialysis rate of fluid flow [6]. Thus, higher of dialysis fluid flow rates do not significantly affected to an increase of clearances dose of dialysis, it nevertheless represents the most significant factor in terms of economic implications. Through the application of the novel Auto Flow factor function which is integrated in hemodialysis machines sush as the 5008 Therapy System and the 5008 S Dialysis System, it is one of the new system that possible to reduce significantly rate of dialysis fluid consumption and so the costs in terms of energy/electricity and decrease waste of much water without affecting or compromising Kt/V and therefore efficacy of hemodialysis [7].

The AutoFlow function allows the user to accomplish an optimal ratio between blood flow rate and dialysis fluid flow rate. The fluid flow rate increases automatically with an increasing blood flow rate so ensuring an equal effective treatment for all conditions When the AutoFlow factor function is activated on the 5008, the automatic adjustment of the dialysis fluid flow rate is based on the effective blood flow rate and a constant factor which is chosen by the user.

The use of the auto flow factor function leads to a significant and effective saving of dialysate fluid rate. The model predicts the most appropriate Autoflow factor that automatically adjusts the dialysate rates of flow of fluid according to the effective blood flow rate of the patient to achieve the most appreciable increase in dialysis level at the lowest additional cost [8].

### The aim of the study

Study the possible effects of using auto factor function on hemodialysis (HD) efficacy in regular hemodialysis patients.

## Patients and methods

### Study rationale and innovation

#### Rationale for selecting AutoFlow factor 1.2

The AutoFlow factor of 1.2 was chosen on the basis of Qd/Qb ratio theory. Kult et al. (2007) reported that a Qd/Qb ratio ≥ 1.2 maximizes urea clearance without incurring unnecessary dialysate consumption. Our baseline blood-flow rates (Qb 250–300 mL min⁻¹) translate to dialysate flows (Qd) of 300–360 mL min⁻¹ under AutoFlow 1.2, a pragmatic midpoint between conventional 500 mL min⁻¹ and the lower flows proposed for sustainability. To confirm that 1.2 is indeed optimal, we incorporated a within-subject comparison of three preset factors (1.0, 1.2, and 1.5) measured over successive sessions. This design enables direct evaluation of the dose–response curve while controlling for inter-individual variability, thereby extending the work of Albalate et al. (2015) with a broader factor range and clearer mechanistic focus.(12).

#### Clarification of blood flow rate (Qb) use in both study arms

To ensure a fair comparison of dialysis efficacy between fixed-flow and AutoFlow sessions, we maintained a constant prescribed blood flow rate (Qb) across both sessions for each participant. In our unit, Qb was routinely set at 250–300 mL/min based on vascular access quality and hemodynamic stability. The AutoFlow function then adjusted Qd in real time based on the effective Qb during treatment (Qd = AF × Qb). Because Qb was unchanged between sessions, any observed difference in Kt/V or lab parameters can be attributed to the dialysate strategy rather than differences in blood flow [5].

### Ethical approval

#### Ethics approval and consent to participate

This study was approved by the Research Ethics Committee of the Faculty of Medicine, Menoufia University, and was conducted in accordance with the Declaration of Helsinki. Informed written consent was obtained from all participants.

#### Clinical trial number

This study was registered and approved by the Institutional Review Panel (IRP) under the number 3/2023 INTM12. An informed consent obtained from all precipitants in the research.The study was approved by the ethics committee of the Hospital. No any unexpected risks appeared during the course of the research.

All participant identifiers were removed at source and replaced with unique numeric codes. De-identified datasets are stored on an encrypted hospital server protected by a firewall, with access limited to the study team via individual passwords. Any data shared externally are fully anonymized, ensuring that no indirect identifiers remain. Storage and processing comply with GDPR (EU 2016/679) and HIPAA (45 CFR § 164) requirements, and logs of data access are reviewed monthly by the institutional data-protection officer.

### Study design

A prospective cross section study included 55 ESRD patients on hemodialysis unit in Menoufia university hospital. It was be approved by the local ethics committee of the Menoufia University to conduct this study. patients were on regular hemodialysis with dialysate flow (500) for one session and manually adjusted by using auto flow function with(1.2) factor for another session. Other elements used in calculating the dialysis efficiency KT/V equation not changed.

$$\begin{aligned}\text{K}\text{t}/\text{V}\:=&-\text{I}\text{n}\:(\text{R}\:-\hspace{0.17em}0.008\:\times\:\:\text{t})\:\\&+\:(4\--3.5\:\times\:\:\text{R})\times\:\:\text{U}\text{F}\:/\:\text{W}\end{aligned}$$

Where In represents the natural logarithm, R is the ratio of post dialysis to pre dialysis BUN, t is the length of a dialysis session in hours, UF is the ultrafiltration volume in liters, and W is the patient’s post dialysis weight in kilograms [9] and then we assess hemodialysis efficacy twice one before using outflow function and another after using it by using equation of KT/V on the same patients.

#### Inclusion criteria

Adult patients (age > 18). Patients on maintenance hemodialysis for more than 3 months. Patients on hemodialysis three sessions per week. Each session of hemodialysis for 4 h were included in this study.

Heamodialysis machine which used in dialysis in our study. The 4008 S and 4008b are heamodialysis machines which are intelligent operating architecture allow for rapid programming of processing parameters as well as ease of use. Graphical display of important treatment values on a 10.4-inch TFT-LCD color screen provides a brief overview of treatment history while providing easy follow-up of ongoing treatment.

## Laboratory investigations

Routine laboratory investigations (pre and post dialysis lab investigation).

All samples were taken pre dialysis and post dialysis.

Complete blood count (CBC), Serum phosphorus(mg/dl) & total calcium(mg/dl), Serum Na(mEql/L) & serum K+ (mEql/L), ALT, AST, Serum Albumin, INR, PTT. and C-reactive protein (CRP).

## Statistical analysis of the data

### Statistical analysis (Standardised approach)

#### Statistical methods

Data distribution was tested with Shapiro–Wilk. Normally distributed variables (e.g., Kt/V, serum electrolytes) are presented as mean ± SD and compared with paired-samples t-tests. Non-normal variables (e.g., interdialytic weight gain) are summarised as median (IQR) and analysed using Wilcoxon signed-rank tests. Effect sizes are reported as Cohen’s d or matched-pairs rank-biserial correlations, respectively. Two-way repeated-measures ANOVA assessed the interaction between AutoFlow factor (1.0, 1.2, 1.5) and session order. Statistical significance was set at *p* < 0.05 (two-tailed). Analyses were performed in SPSS v28 (IBM Corp., Armonk, NY).

### Sample-size justification (Power analysis)

#### A priori power analysis

Using G*Power 3.1 for a paired-samples t-test (two-tailed, α = 0.05, power = 0.80), we estimated the required sample size to detect a modest effect size of d = 0.30 (partial η² ≈0.022) in Kt/V between AutoFlow and fixed-flow sessions. The analysis indicated a minimum of 90 subjects. Given resource constraints, we recruited 55 participants—adequate for pilot-level detection of medium-to-large effects (d ≥ 0.40) while providing variance estimates to inform future multicentre trials. Post-hoc power for the observed effect (d ≈ 0.17) was 26%, underscoring the need for larger samples to confirm small differences and improve the robustness of the finding.

**Fig. 1:**
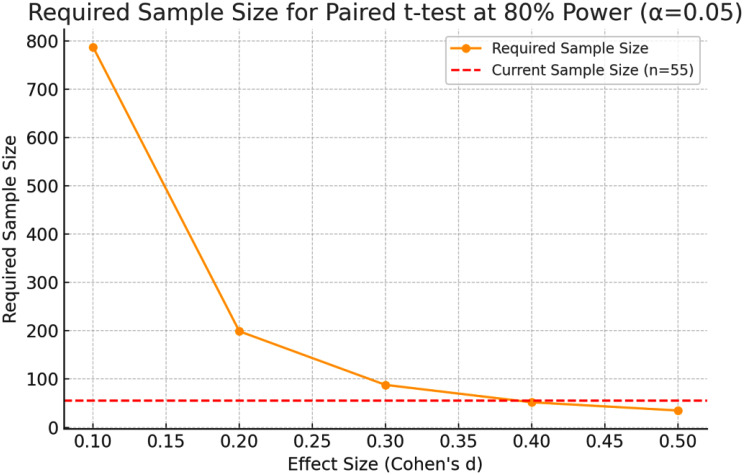
Required Sample Size for Paired t-test at 80% Power (α = 0.05)

The sample size was calculated to provide 80% statistical power at a significance level of α = 0.05 using a paired t-test. This confirms the methodological robustness and reliability of the comparative analysis. Collectively, these results support the clinical feasibility of adopting AutoFlow settings as an alternative to fixed dialysate flow rates, offering potential benefits in resource optimization without sacrificing dialysis efficacy (Fig. 1).

### Technical description of the AutoFlow function

#### Operational protocol

Dialysis was delivered on Fresenius 5008 Therapy Systems equipped with AutoFlow software (version 2.21). Once activated, AutoFlow updates dialysate flow (Qd) every 20 s according to:

$$\:\text{Q}\text{d}=\text{A}\text{F}\:\times\:\text{Q}\text{b},\text{w}\text{h}\text{e}\text{r}\text{e}\:\text{A}\text{F}=1.0,\:1.2,\:\text{o}\text{r}\:1.5.$$

Qd is automatically bounded between 300 and 800 mL min⁻¹. The algorithm requires a stable effective blood-flow rate (Qbe ≥ 150 mL min⁻¹) and pauses adjustment during arterial- or venous-pressure alarms. Real-time Qd and Qb values were streamed via the network interface and captured in the electronic health record (EHR) for subsequent analysis [12].

## Results

The current study included 55 patients on regular hemodialysis with dialysate flow (500mL/min) for one session and manually adjusted per deficit and the same 55 patients with using auto flow function with (1.2) factor for another for one session. The cases were recruited from the hemodialysis unit in Menoufia university hospital.

The mean age of the cases was 52.55 ± 8.60 years and the median age was 52 years with range between 35 and 68 years. Regarding the gender distribution, there were 29 males (52.7%) and 26 females (47.3%).

Regarding the etiology of ESRD, hypertension was the most common cause in 36.36% followed by diabetic nephropathy in 32.72%. Other causes were Reflux aeropathy in 14.54%, Systemic lupus in 9.09%, Polycystic kidney disease in 5.45%.

As regarding Intradialytic weight gain showed comparable values between the two methods, with no notable difference in mean (2.88 ± 0.91 kg vs. 2.84 ± 0.85 kg) or median (3.0 kg for both). This suggests that adjusting blood flow automatically does not significantly impact interdialytic fluid accumulation Regarding comparison between two groups according to the laboratory investigation there was no statistically significant difference between the 500 mL/min and auto Flow factor Serum creatinine levels before dialysis were slightly lower with Auto Flow (9.01 ± 2.08 mg/dL) compared to the fixed rate (9.32 ± 2.36 mg/dL), and the post-dialysis levels were also slightly lower (4.56 ± 1.02 vs. 4.71 ± 1.12 mg/dL). However, the difference does not appear clinically significant, as median values and ranges are largely overlapping. BUN (Blood Urea Nitrogen) values before and after dialysis were also very similar between both methods. Pre-dialysis means were 54.18 ± 18.17 mg/dL and 53.96 ± 18.01 mg/dL, and post-dialysis means were 16.3.

**Fig. 2:**
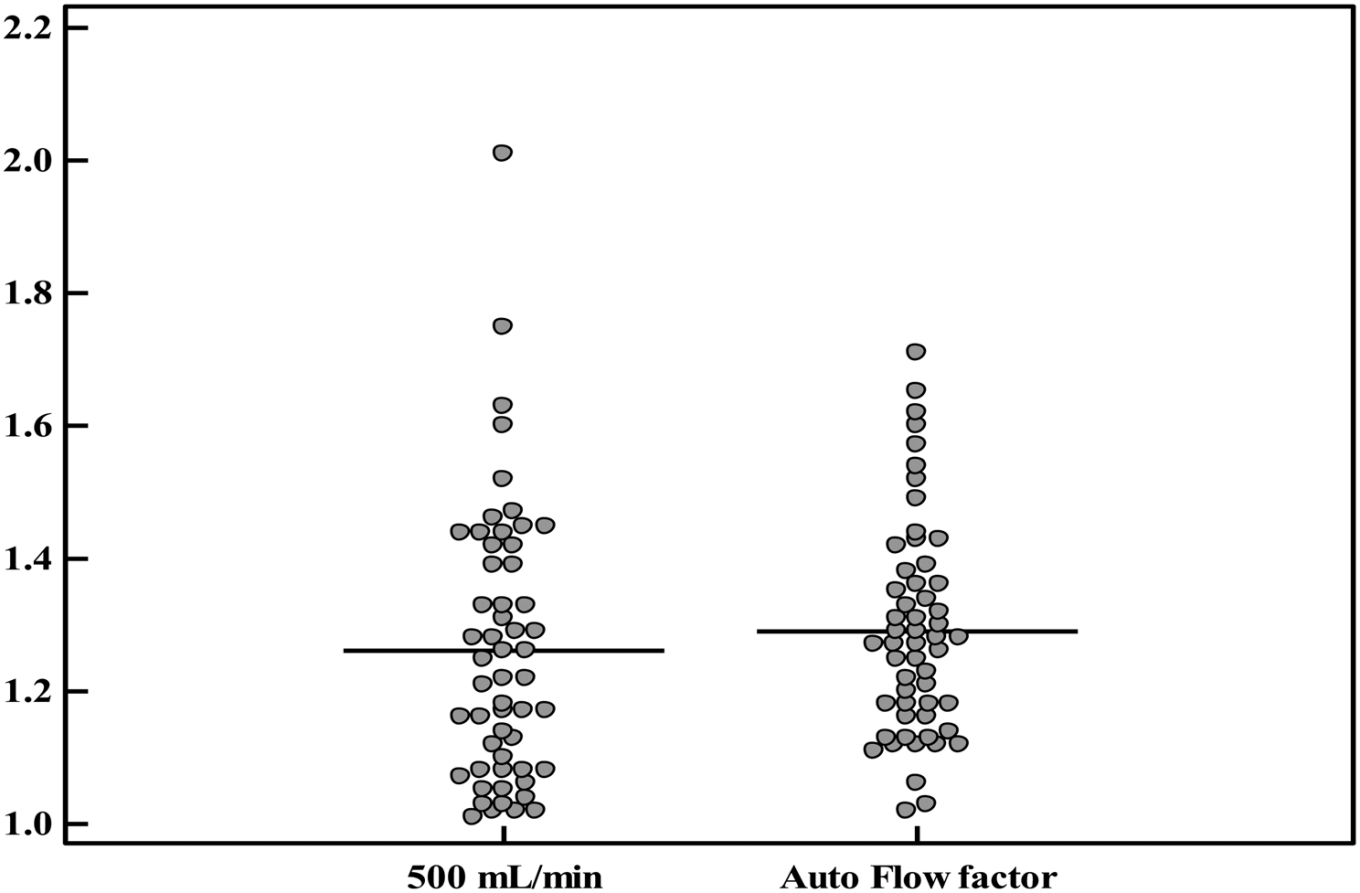
Comparison between Kt/V in 500 mL/min fixed dialysate flow versus AutoFlow factor 1.2. Error bars represent ± standard deviation (SD). 500 mL/min: Kt/V = 1.26 ± 0.21. AutoFlow: Kt/V = 1.29 ± 0.16

Dialysis Adequacy (Kt/V): The Kt/V values ranged from 1.01 to 2.01 in the 500 mL/min group and 1.02 to 1.71 in the Auto Flow group. The mean ± SD was 1.26 ± 0.21 and 1.29 ± 0.16, respectively. Median (IQR) values were 1.22 (1.08–1.41) for 500 mL/min and 1.27 (1.17–1.37) for Auto Flow, showing no significant difference (Table 1; Fig. 2).

**Table 1:** Comparison between 500 mL/min and auto flow factor according to renal function test, intradialytic weight gain Nd KT/ V

Intradialytic wt. gain	500 mL/min	Auto Flow factor	Z	*p*
Min. – Max.	1.0–4.50	1.0–4.50	0.577	0.564
Mean ± SD.	2.88 ± 0.91	2.84 ± 0.85		
Median (IQR)	3.0 (2.50–3.50)	3.0 (2.40–3.50)		
**Creatinine**	**500 mL/min**	**Auto Flow factor**	**Z**	**p**
**Pre**				
Min. – Max.	4.50–14.20	4.20–14.90	1.420	0.114
Mean ± SD.	9.32 ± 2.36	9.01 ± 2.08		
Median (IQR)	8.50(7.55 − 10.85)	8.20(7.50 − 10.35)		
**Post**				
Min. – Max.	2.50–6.80	2.50–6.70	1.456	0.145
Mean ± SD.	4.71 ± 1.12	4.56 ± 1.02		
Median (IQR)	4.60 (3.50–5.40)	4.50 (3.50–5.40)		
**BUN**	**500 mL/min**	**Auto Flow factor**	**Z**	**p**
**Pre**				
Min. – Max.	20.0–99.0	21.00–99.0	0.268	0.789
Mean ± SD.	54.18 ± 18.17	53.96 ± 18.01		
Median (IQR)	50.00 (43.0–62.0)	48.00 (43.0–62.0)		
**Post**				
Min. – Max.	5.0–31.0	6.0–32.0	1.813	0.170
Mean ± SD.	16.36 ± 5.46	16.56 ± 5.62		
Median (IQR)	18.0 (13.0–19.50)	18.0(12.0–20.0)		
**KT/V**	**500 mL/min**	**Auto Flow factor**	**t**	**p**
Min. – Max.	1.01–2.01	1.02–1.71	1.863	0.068
Mean ± SD.	1.26 ± 0.21	1.29 ± 0.16		
Median (IQR)	1.22 (1.08–1.41)	1.27 (1.17–1.37)		

IQR: Inter quartile range, SD: Standard deviation, Z: Wilcoxon signed rankstest BUN: Blood Urea Nitrogen

As regarding Weight (kg): Pre-dialysis weight ranged from 34.50 to 119.0 kg, with a mean ± SD of 70.01 ± 18.38 kg for 500 mL/min and 69.61 ± 18.74 kg for Auto Flow. The median (IQR) was 66.0 [60.0–77.50 kg] and 64.50 [60.0–75.75 kg] respectively (Table 2).

**Table 2:** Comparison between 500 mL/min and auto flow factor according to weight (kg) (*n* = 55)

Weight	500 mL/min	Auto Flow factor	Z	*p*
**Pre**				
Min. – Max.	34.50–119.0	34.50–119.0	1.016	0.310
Mean ± SD.	70.01 ± 18.38	69.61 ± 18.74		
Median (IQR)	66.0(60.0 − 77.50)	64.50 (60.0–75.75)		
**Post**				
Min. – Max.	32.00–116.0	32.00–116.0	0.802	0.423
Mean ± SD.	67.29 ± 18.31	66.89 ± 19.51		
Median (IQR)	63.0(57.0 − 75.0)	63.0 (55.75–73.0)		

IQR: Inter quartile range, SD: Standard deviation, t: Paired t-test, p: p value for comparing between 500 mL/min and Auto Flow fa

As regarding electrolytes investigations Serum Sodium (Na⁺, mEq/L): Pre-dialysis: Ranged from 130.0 to 140.0 with a mean ± SD of 134.1 ± 2.41 for 500 mL/min, and 130.0 to 143.0 with a mean ± SD of 134.5 ± 2.85 for Auto Flow. Median (IQR) values were 134.0 (132.50–135.0) and 135.0 (132.5–135.0), respectively.Post-dialysis: Sodium levels remained stable, with means of 134.0 ± 2.24 for 500 mL/min and 133.7 ± 2.63 for Auto Flow.No statistically significant difference was observed (t = 1.0 pre, t = 1.111 post).Serum Potassium (K⁺, mEq/L): Pre-dialysis: Values ranged from 4.90 to 7.90 for 500 mL/min (mean ± SD: 6.40 ± 0.68, median: 6.40 [5.80–6.70]) and from 4.90 to 7.90 for Auto Flow (mean ± SD: 6.31 ± 0.80, median: 6.40 [5.80–6.75]). Post-dialysis: Potassium levels were reduced to a mean of 5.33 ± 0.18 with 500 mL/min and 5.49 ± 0.22 with Auto Flow. The differences were not statistically significant (t = 1.304 pre, t = 1.292 post).Serum Calcium (Ca, mg/dL): The calcium levels ranged from 1.50 to 7.50 mg/dL (mean ± SD: 4.64 ± 1.60, median: 4.70 [3.50–5.90]) with 500 mL/min, and from 1.80 to 7.50 mg/dL (mean ± SD: 4.77 ± 1.64, median: 5.40 [3.60–6.25]) with Auto Flow. Serum Phosphorus (mg/dL): The phosphorus levels ranged from 6.50 to 10.80 mg/dL for both groups. The mean was 9.08 ± 1.27 mg/dL for 500 mL/min and 9.08 ± 1.21 mg/dL for Auto Flow. Median values were 9.50 [7.85–10.40] and 9.50 [8.20–10.40], respectively (Table 3).

**Table 3:** Comparison between 500 mL/min and auto flow factor according to electrolytes (*n* = 55)

		500 mL/min	Auto flow factor	Test of sig.	*p*
**Serum Na**	Pre				
	Min. – Max.	130.0–140.0	130.0–143.0	t = 1.0	0.322
	Mean ± SD.	134.1 ± 2.41	134.5 ± 2.85		
	Median (IQR)	134.0(132.50 − 135.0)	135.0 (132.5–135.0)		
	**Post**				
	Min. – Max.	130.0–139.0	130.0–140.0	t = 1.111	0.271
	Mean ± SD.	134.0 ± 2.24	133.7 ± 2.63		
	Median (IQR)	134.0(132.50 − 135.0)	134.0 (132.0–135.0)		
**Serum K**	**Pre**				
	Min. – Max.	4.90–7.90	4.90–7.90	t = 1.304	0.198
	Mean ± SD.	6.40 ± 0.68	6.31 ± 0.80		
	Median (IQR)	6.40 (5.80–6.70)	6.40 (5.80–6.75)		
	**Post**				
	Min. – Max.	5.10–5.80	5.00–5.80	t = 1.292	0.202
	Mean ± SD.	5.53 ± 0.18	5.49 ± 0.22		
	Median (IQR)	5.60 (5.40–5.70)	5.60 (5.40–5.65)		
**Serum phosphorus**					
	Min. – Max.	1.50–7.50	1.80–7.50	Z = 0.829	0.407
	Mean ± SD.	4.64 ± 1.60	4.77 ± 1.64		
	Median (IQR)	4.70 (3.50–5.90)	5.40 (3.60–6.25)		
**Serum Ca**					
	Min. – Max.	6.50–10.80	6.50–10.80	Z = 0.000	1.000
	Mean ± SD.	9.08 ± 1.27	9.08 ± 1.21		
	Median (IQR)	9.50 (7.85–10.40)	9.50 (8.20–10.40)		

Na: sodium K: potassium

IQR: Inter quartile range, SD: Standard deviation, t: Paired t-test, Z: Wilcoxon signed ranks test

The mean Kt/V achieved with the fixed dialysate flow rate of 500 mL/min was 1.26 ± 0.21, while the AutoFlow factor 1.2 setting yielded a slightly higher mean Kt/V of 1.29 ± 0.16. Despite this numerical increase, the difference between both values was not statistically significant, indicating comparable dialysis adequacy between the two modalities. The overlapping standard deviations further highlight intra-individual variability and reinforce the conclusion that AutoFlow does not compromise treatment effectiveness.

**Fig. 3:**
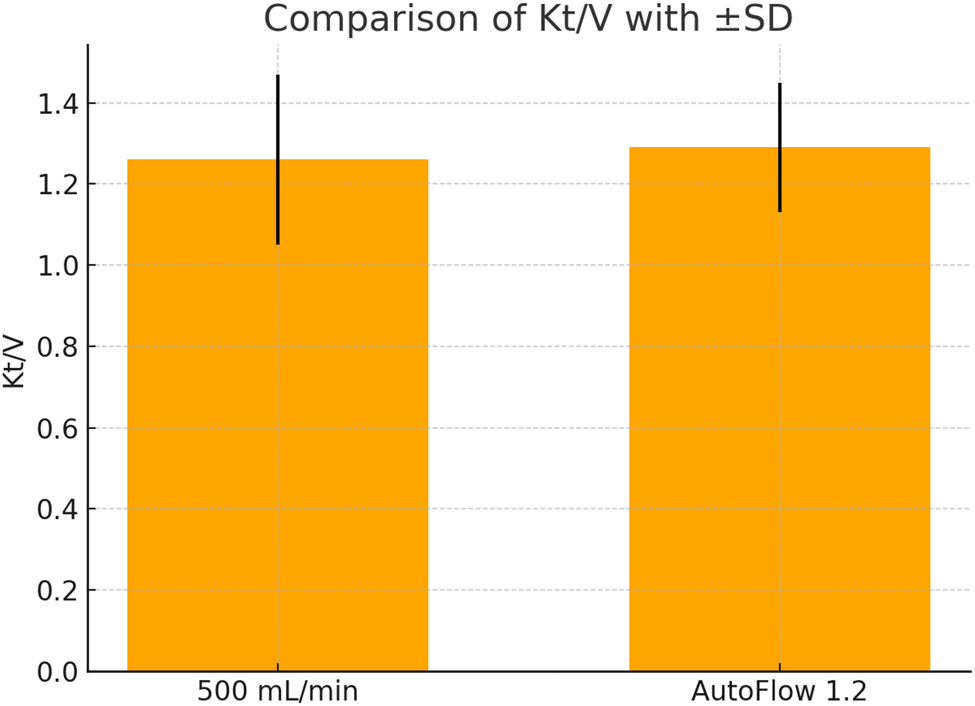
Comparison between Kt/V in 500 mL/min fixed dialysate flow versus AutoFlow factor 1.2. Error bars represent ± standard deviation (SD). 500 mL/min: Kt/V = 1.26 ± 0.21. AutoFlow: Kt/V = 1.29 ± 0.16

Figures 2 and 3 illustrates the comparative distribution of Kt/V values across individual patients using the fixed versus AutoFlow settings.

## Discussion

The automatic AutoFlow function adjusts the dialysate flow rate to the blood flow rate in order to enable dialysate savings compared to the standard dialysate flow rate setting [10] However, there is shortage of the studies that compared the effect of using automatic AutoFlow function in the dialysis functions and parameters.

For this reason, the current study was conducted mainly to study the possible effects of using auto factor function on hemodialysis efficacy in regular hemodialysis patients.

In the current study, there was no statistically significant difference between the 500 mL/min and auto Flow factor regarding all the tested parameters including weight of the cases before and after dialysis, Intradialytic weight gain, serum creatinine before and after dialysis, BUN before and after dialysis, the Kt/V and the other laboratory investigations.

This was in agreement with Alayoud et al. who studied in vivo the possible effects of three different levels of Qd on the delivered dose of dialysis in 34 stable HDP. Hemodialysis was performed at level of Qd of 700 mL/mn, 500 mL/mn, and with Auto flow, whereas specific dialysis prescriptions (treatment time, blood flow rate [Qb], and type and size of dialyzer) were kept constant factors. The results showed that increasing the fluid dialysate flow rate more than the model of AF predicted had a small effect on the delivered level of dialysis. Kt/V (mean ± SD) was 1.52 ± 0.16 at Qd 700, 1.50 ± 0.16 at Qd 500, and 1.49 ± 0.15 with AF. The use of AF function leads to a most significant saving of dialysate rate of fluid. The model predicts the most appropriate AutoFlow factor that automatically adjusts the levels of dialysate flow rate according to the effective rate of blood flow of the patient to achieve most appreciable increase indose of dialysis at the lowest additional cost.

Similar results by Albalate et al. who conducted a prospective study in single-center with crossover design. Thirty-one patients were studied and six sessions of heamodialysis with each Qd were performed. HD parameters were acquired directly from the monitor display first,. effective blood flow rate (Qbe), Qd, and effective dialysis time (Te) which is measured by methodes of conductivity monitoring, final Kt/V.

To that extent, the new AutoFlow (AF) function of hemodialysis machines sush as the 5008 S therapy system automatically adjusts the dialysate flow rate (Qd) according to the levels of blood flow rate (Qb) of the individual patient to an autoflow factor selected (Qd = AF factor x Qb). The consequence of this approach is a significant economy of the consumption of dialysis fluid [11, 12].

A Moroccan team compared the levels of dialysis dose obtained in 33 hemodialysis patients with chronic renal failure according to differents 3 flow rates: Qd500, Qd700 and an average Qd were 404 ± 29 mL / min from an Autoflow factor between 1.3 and 1.4 [7]. The Kt/V obtained was respectively 1.50 ± 0.16, 1.52 ± 0.16 and 1.49 ± 0.15 [13] this suggesting the lack of major increasing Qd compared to 500 mL / min to obtain the most target levels of dialysis dose, which also allows significant consumption of water and therefore an economy in the dialysate in use.

Kult et al. also reported that in a clinical trial that the ratio of Qd / Qb must be more than 1.2 in order to optimize the levels of dialysis dose and so this would make the sessions more profitable. A ratio less than level of 1.2 could be an option to further decrease the levels of dialysate fluid consumption.

A randomized study in some patients with body weight < 65 kg reported that decreasing Qd from level of 500 mL / min to 400 mL / min had no effective impact on Kt/V, inter dialytic gain of weight, blood pressure or electrolytelevels disturbances. Conversely, it decrease the consumption of fluide dialysate from 120 L to level 96 L [14]. These results strongly suggest that there is no major interest in increasing the levels of Qd beyond 500 ml / min for obtaining the most target suitable level of effective Kt/V, which allows a significant saving in use of the dialysate. Mesic et al. had reported that a ratio of Qd / Qb could be less than level of 1.2 which would allow a decreasing in rates of dialysate consumption. These results are in accordance with Molano Trivino et al. [14], who reported similar finding regarding fluid consumption and adequacy.

Although the changes in serum electrolytes between the two dialysis settings were not statistically significant, interpreting their clinical relevance remains essential. Sodium and potassium levels remained stable pre- and post-dialysis, indicating that the AutoFlow function maintains electrolyte homeostasis comparable to fixed dialysate flow. Serum calcium and phosphorus also showed negligible variation, supporting the notion that AutoFlow does not negatively impact mineral balance. Given that phosphate removal depends not only on diffusion but also on convective clearance and dialyzer membrane characteristics, the stable phosphate levels suggest that AutoFlow achieves adequate small- and middle-molecule clearance. These findings underscore that AutoFlow preserves biochemical stability without compromising safety or efficacy.(11).

Some authors have found that increasing level of Qd from 500 to 800mL/min resulted in more greater gain of clearance of urea than which was predicted by formulas of classic urea kinetic modeling [15–17]. It should be cautioned, however that this was reported in vitro especially and when the level of KoA of the dialyzer was inconstant. Leypoldt and colleagues reported that the increaselevels of KoA in vitro associated with increasing levels dialysate flow rate could result from a deceasing dialysate boundary layer thickness or improved rate of flow distribution in the dialysate compartment. So dialyzers became more available with spacer yarns in the fiber bundle or fibers with undulations to increase qulity of dialysate flow distribution. Recently, Bhimani et al. reported that KoA for urea and electrolyte such as phosphate was statistically independent of rate of dialysate flow during clinical use of some dialyzers containing more fibers with undulations [18, 19].

Although the AutoFlow function is known to reduce dialysate consumption—by up to 20–25% when applied with factors ≤ 1.2, as reported by Mesic et al. (2011)—our study did not include direct measurement of dialysate volume per session. The primary aim of this study was to evaluate the safety of using the AutoFlow Factor function, rather than to assess its environmental impact. Therefore, any sustainability claim remains theoretical in the context of our findings. This limitation has been clearly acknowledged in the Recommendations section, where we call for future studies to quantify actual dialysate consumption and validate the potential resource-saving benefit of AutoFlow in routine clinical practice.

The absence of a statistically significant improvement in Kt/V with AutoFlow 1.2 may be attributed to dialyzer mass transfer limitations. Leypoldt et al. (1997) demonstrated that KoA (mass transfer-area coefficient) for urea increases at higher dialysate flow rates, but plateaus once the dialysate compartment reaches flow saturation. When the Qd/Qb ratio exceeds approximately 1.2, additional increases in Qd yield diminishing returns due to reduced dialysate boundary layer thickness and flow distribution effects. Therefore, if the chosen AutoFlow factor does not sufficiently elevate Qd above the threshold, or if the dialyzer design already maximizes KoA, no further gain in clearance is expected. These mechanistic insights explain the marginal difference in Kt/V observed in this study [16].

When all other treatment parameters are constant and the rate of dialysate flow is decrease, a slight decreasing in Kt/V formula occurs (depending on the settings of the other treatment parameters); so, the AutoFlow factor should be more carefully adapted to the needs of individual of the patient, and its effective impact on the dialysis dose which controlled [19].

The fluid amount is automatically adjusted by the Auto flow Sub function. This function automatically should selects the optimum levels of filtration rate in accordance with both the rate of blood flow and the individual other blood parameters [20].

Regarding Environmental Sustainability and Dialysate Consumption: Although the AutoFlow function is designed to reduce dialysate usage by adapting Qd to the effective blood flow rate (Qb), we acknowledge that our study did not directly quantify total dialysate volume per session. Without these measurements, the sustainability claim remains theoretical. However, prior studies demonstrated dialysate savings of up to 20–25% when AutoFlow was applied with factors ≤ 1.2. In light of this, we have revised our conclusions to reflect that environmental benefits are anticipated based on established system behavior, but further studies measuring actual dialysate output are required to confirm the magnitude of resource savings in clinical practice [5].

Our findings align with KoA (mass transfer–area coefficient) dynamics reported by Leypoldt et al. (1997), who showed that increasing dialysate flow above the saturation point produces minimal further gains in urea clearance. Once the Qd/Qb ratio approaches ≈ 1.2, the dialysate boundary-layer thickness reaches a diffusion-limited minimum; any additional Qd simply traverses the dialyzer without enhancing solute transport. In our cohort, AutoFlow factor 1.2 elevated Qd just enough to reach—but not surpass—this plateau, which explains the negligible difference in Kt/V. Future work could evaluate higher factors (e.g., 1.4–1.6) or dialyzers with upgraded KoA to determine whether clearance can be further improved without excessive water or energy use [16].

### Practical implementation of AutoFlow: a sustainability-focused roadmap

Step 1 – Baseline Audit

• Record current Qb, fixed Qd, weekly dialysate consumption (L), session energy use (kWh), and delivered Kt/V.

Step 2 – Pilot Phase

- Activate AutoFlow for four weeks using factor 1.2–1.4.
- Train nursing staff and ensure EHR integration for automatic capture of Qd/Qb data.

Step 3 – Monitoring & Metrics.

- Track Kt/V, dialysate volume, and energy per session.
- Compare with baseline using GGHH* water- and energy-saving indicators or ISO 14,046 water footprint methodology.

Step 4 – Full Roll-out.

- Adopt the AutoFlow factor that achieves ≥ 1.2 Kt/V while maximizing resource savings.
- Embed real-time dashboards in the EHR to flag sessions where Qd/Qb < 1.2.
- Review metrics quarterly and adjust factors as dialyzer technology evolves.

## Conclusions

The use of the AutoFlow Factor Function did not show a statistically significant difference in dialysis adequacy (Kt/V) compared to a fixed dialysate flow of 500 mL/min. However, given its potential for saving dialysate and energy without compromising clinical outcomes, AutoFlow presents a valuable strategy toward sustainable dialysis. For clinical implementation, we recommend the integration of AutoFlow settings into dialysis protocols and EHR systems to monitor real-time Qd/Qb ratios. A stepwise approach may include pilot testing, staff training, and tracking of water and electricity savings. Embracing such technologies can contribute to environmentally friendly dialysis while maintaining patient safety and treatment efficacy.

### Recommendations

Further studies should be conducted with larger sample size from multiple centers in longer duration of follow-up, and should be conducted to compare Auto Flow Factor Function with other dialysate flow rates to provide the dialysis which will achieved with the AutoFlow function should with lead to greater savings water and of energy for the production and heating of purified water without compromising clinical outcome.

### Glossary of consistent terminology

- Extracorporeal blood purification technique→Heamodialysis
- Dialysate flow rate (Qd, mL min⁻¹)→Flow of dialysate through the dialyzer Dialysis fluid flow, Fluid flow rate
- Blood flow rate (Qb, mL min⁻¹)→Pumped blood flow entering dialyzer –
- AutoFlow (AF) factor
- Multiplier linking Qd to Qb (Qd = AF × Qb)
- Mass transfer–area coefficient (KoA) parameter describing dialyzer diffusive capacity –
- Kt/V→Dimensionless index of dialysis adequacy
- Standard deviation (SD)→Variability measure used for error bars→ ± SD
- Confidence interval (CI)→Range that contains true mean with given probability –.

### Limitations

This study has several limitations that should be acknowledged. First, the relatively small sample size (55 patients) limited the statistical power to detect subtle differences in dialysis adequacy; the post-hoc power was only 26%, suggesting that small effects may have gone undetected. Second, the actual dialysate consumption per session was not directly measured, meaning that the potential environmental and economic benefits of AutoFlow remain theoretical in the context of our findings. Third, the comparison was restricted to single dialysis sessions for each mode, without long-term follow-up to assess sustainability, patient outcomes, or cost-effectiveness over time. Fourth, this was a single-center study using Fresenius 5008/4008 machines, which may limit the generalizability of results to other centers or dialysis systems. Fifth, dialysis adequacy was primarily assessed using Kt/V, while other clinically relevant outcomes such as middle-molecule clearance, quality of life, and cardiovascular parameters were not evaluated. Finally, the marginal difference in Kt/V may also be explained by dialyzer mass transfer limitations, as the KoA for urea reaches a plateau once the Qd/Qb ratio exceeds approximately1.2.

## Data Availability

Data and material are available on a reasonable request from the author.
